# Angiogenesis in Acute Myeloid Leukemia and Opportunities for Novel Therapies

**DOI:** 10.1155/2012/128608

**Published:** 2011-09-05

**Authors:** Angelica Trujillo, Christie McGee, Christopher R. Cogle

**Affiliations:** Division of Hematology and Oncology, Department of Medicine, College of Medicine, University of Florida, Gainesville, FL 32610-0278, USA

## Abstract

Acute myeloid leukemia (AML) arises from neoplastic transformation of hematopoietic stem and progenitor cells, and relapsed disease remains one of the greater challenges in treating this hematologic malignancy. This paper focuses on angiogenic aspects of AML including the significance and prognostic value of bone marrow microvessel density and circulating cytokine levels. We show three general mechanisms whereby AML exploits angiogenic pathways, including direct induction of angiogenesis, paracrine regulation, and autocrine stimulation. We also present early evidence that leukemia cells contribute directly to vascular endothelia. Novel treatment strategies are proposed, and a review of relevant antiangiogenic clinical trials is presented. By understanding how blood vessels can serve as a reservoir for refractory and relapsed AML, new diagnostics and promising treatment strategies can be developed.

## 1. Introduction

Acute myeloid leukemia (AML) is a cancer of the bone marrow characterized by a mutation in a hematopoietic stem or progenitor cell (HSPC), which develops into a highly proliferative accumulation of dysfunctional and immature myeloid cells. These abnormal cells eventually dominate hematopoietic niches like the bone marrow and result in abnormal peripheral blood counts (anemia, thrombocytopenia, leukocytosis due to high number of myeloblasts number and/or neutropenia) [1]. Initial disease remission can be achieved in 30–70% of AML patients after standard induction chemotherapy regimens such as 7 + 3 (seven days of cytarabine and 3 days of an anthracycline). However, refractory and relapsed disease remains a major challenge in all patients, especially in older individuals. In patients 60 years and older, the 5-year survival prognosis for AML is only 20% with the majority of patients succumbing to disease relapse [2, 3]. Mechanisms for AML relapse are related to leukemia cell insensitivity and potential sanctuary sites. When considering that functional blood vessel networks are essential to these mechanisms of relapse, the investigation of angiogenesis in leukemia is highly significant. 

Until recently, leukemia studies have focused primarily on the leukemia cell. However, with mounting evidence showing the importance of the bone marrow microenvironment in regulating hematopoiesis, it is necessary to broaden the scope of investigation beyond the leukemia cell. A better understanding of the pathobiology surrounding and supporting leukemia cell survival has great potential to lead to promising new therapies.

## 2. Endothelial Cells in Support of Leukemia

The importance of the cancer microenvironment is widely recognized in solid tumors. Cancer cells interact with the stromal microenvironment in complex ways to promote their own survival and proliferation. However, in the case of hematologic malignancies like AML, the leukemia microenvironment is highly dynamic. The typical leukemia niche is within the bone marrow microenvironment. But AML cells can also migrate systemically to other organs that support hematopoiesis, such as the liver and spleen [4]. Monocytic AML subtypes (M4 and M5 FAB subtypes) can also migrate across blood-organ barriers and into privileged areas such as the central nervous system. 

**Figure 1:**
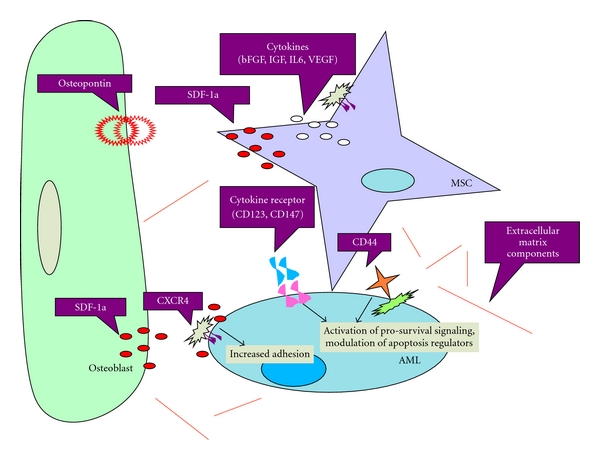
Acute Myeloid Leukemia Cells Within the Bone Marrow Microenvironment. The bone marrow niche can be simplistically divided into the endosteal niche or osteoblastic niche which is located on the inner bone surface. Hematopoietic stem cells (HSCs) have been found to reside here in a quiescent state. The vascular niche is made up a central sinusoid and lined by endothelial cells, macrophages, and perivascular cells. In the central marrow region, between the endosteal niche and vascular niche, acute myeloid leukemia (AML) cells hijiack the entire bone marrow anatomy and induce angiogenesis. The location of AML initiation and relapse within the bone marrow has yet to be defined.

Although the complete model of the bone marrow microenvironment is not yet fully understood, it has been simplistically divided into three compartments: an endosteal niche that maintains quiescent hematopoietic stem cells, a vascular niche which regulates entry and exit from the bone marrow, and the central marrow space filled with various hematopoietic progenitors in the process of differentiation [5] (Figure 1). 

One of the earlier investigations in the relationship between AML cells and the vascular niche was performed by Fiedler et al. [6]. These investigators found that a large proportion of AML patients had disease that expressed vascular endothelial growth factor (VEGF), as well as VEGFR1 and VEGFR2. They also found that VEGF induced human umbilical vein endothelial cells (HUVECs) secrete GM-CSF, which is a known mitogen for AML cells. Together, these results were one of the first to suggest that AML cells (i) exploit angiogenic signaling for autocrine stimulation and (ii) provoke endothelial cells to secrete proleukemic factors for survival and proliferation.

Recent evidence indicates that leukemia cells, like tumor cells, depend on angiogenesis in the bone marrow. Clinically, increased angiogenesis has been reported in the bone marrow of patients with AML. Hussong et al. stained bone marrow biopsies for blood vessels in 20 patients with untreated AML, compared with 20 control patients and quantified the number of vessels/mm in each case [7]. They found significantly increased microvessel density (MVD) in the bone marrow of AML patients (*P* < 0.001), suggesting a role of angiogenesis in AML. This is particularly significant when considering the strong positive correlation between increased bone marrow vasculature and overall survival of leukemia [8, 9]. A higher microvessel density predicted for poor prognosis and suggests that blood vessel-AML interactions may contribute to refractory disease [10]. 

**Figure 2:**
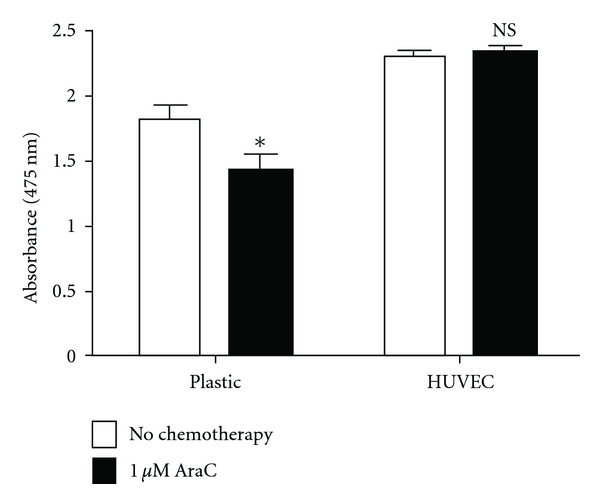
Endothelial Cells Protect Acute Myeloid Leukemia Cells from Chemotherapy. Human acute promyelocytic leukemia cells (HL60) were cultured in two conditions: over plastic and in the presence of human umbilical vein endothelial cells (HUVECs). The leukemia cells were then exposed to cytarabine chemotherapy, which is commonly administered to patients with AML. Cell proliferation was subsequently measured by XTT assay. HL60 cells in coculture with HUVECs showed no decrease in cell proliferation after chemotherapy exposure (NS) as compared to HL60 cells cultured over plastic (*P* < 0.05).

Endothelial cells support adhesion and transmigration of subsets of normal CD34+ HSPCs. *In vitro* studies have shown that upon transwell or direct coculture with HUVECs, AML blasts proliferate to a higher degree and are less susceptible to traditional chemotherapeutic agents such as cytarabine [11]. We have found similar results when coculturing human promyelocytic leukemia cells (HL60) with HUVECs and then exposing the cells to cytarabine. HUVECs protect AML cells from chemotherapy (Figure 2). Together, these results provide compelling evidence that endothelial cells are protective of leukemia and may be a site of leukemia reinitiation after chemotherapy. Although the protective effect of endothelial cells on leukemic myeloblasts is evident, a thorough understanding of the detailed interactions and mechanisms is necessary in order to rationally design new therapeutic strategies.

While endothelial cells enhance leukemia proliferation, emerging evidence indicates that leukemia cells may have a reciprocal effect of enhancing endothelial cell proliferation. Hatfield et al. investigated the hypothesis of interdependence using transwell and direct contact experiments between primary AML cells and dermal microvascular endothelial cells (DMVECs). ^3^H-Thymidine incorporation assays were used to quantify proliferation of endothelial cells. Their results showed enhanced endothelial cell proliferation in transwell coculture with AML blasts [12]. At the very least, this codependence is mediated by secreted cytokines between leukemia cells and endothelial cells. Cytokines that are involved in this bidirectional crosstalk include, for example, VEGF, angiopoietins, GM-CSF, CXCL8, and IL-6. A study by Kruizinga et al. [13], evaluated the leukemia cell expression levels of several VEGF isoforms in a pediatric AML population. The study used PCR arrays to analyze AML myeloblast cells for the presence of the cytokines. Various isoforms of VEGF including VEGF-121, VEGF-165, and VEGF-189 were expressed in the AML cells. Particularly, the VEGF-165 and VEGF-189 isoforms stimulated endothelial proliferation and angiogenesis. However, correlations between VEGF mRNA isoform expression levels and known prognostic factors were not found, nor was there a relationship between VEGF expression and overall survival or relapse-free survival. 

**Figure 3:**
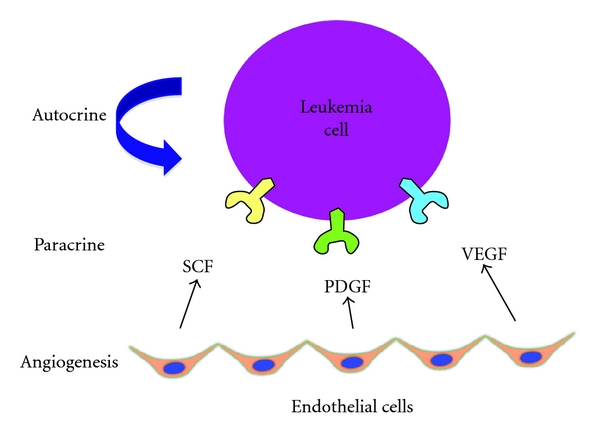
Multiple Mechanisms of Angiogenic Pathways Regulate Acute Myleoid Leukemia Survival and Proliferation. Acute myeloid leukemia cells exploit angiogenic mechanisms by (1) inducing angiogenesis directly, (2) expressing receptors for specific angiogenic growth factors (paracrine regulation), and (3) secreting their own angiogenic factors for their own angiogenic growth factor receptors (autocrine stimulation). Thus, angiogenesis has both cell-extrinsic and cell-intrinsic significance in leukemia. Stem cell factor (SCF), platelet-derived growth factor (PDGF), and vascular endothelial growth factor (VEGF) are a few of many yet to be defined angiogenic factors that regulate leukemia cell survival and proliferation.

With ECs promoting AML cells, and AML cells promoting ECs, a cyclical positive feedback loop is established and strongly favors the potential for refractory and relapsed disease (Figure 3). 

Clinically, increased levels of circulating angiogenic factors correlate with increased angiogenesis in the bone marrow [14] and are high-risk indicators of disease relapse and early mortality [15]. Some of these circulating pro-angiogenic factors include VEGF and the angiopoietins. The clinical relevance of other angiogenic mediators, such as basic fibroblast growth factor (bFGF) and IL-6, has yet to be defined. The presence of angiogenic mediators in leukemia is complicated by the variabilities of gene expression and factor secretion. Cytokine levels vary on a patient-by-patient (disease-by-disease) basis, but evaluating the presence of these cytokines can be used as a prognostic indicator. Hou et al. [16] assessed the expression of a few of these cytokines in mononuclear cells of the bone marrow in 126 newly diagnosed AML patients prior to treatment to correlate prognostic outcome. By PCR screening, they found that a high level of pretreatment angiopoietin-2 (Ang-2) was a prognostic indicator of poor outcome. There is contrary evidence from others [17] that showed that pretreatment levels of Ang-2 were a prognostic indicator of good clinical outcomes. However, in common between these seemingly conflicting reports were the observations that in the presence of high VEGF-A levels, high Ang-2 correlated with poor outcome. Thus, the significance of Ang-2 in AML is complex and most likely influenced by VEGF activity. The angiopoietin/Tie2 axis may be important in AML and certainly needs further definition.

## 3. Leukemia Cells with Endothelial Cell-Like Phenotype and Function

The ability of leukemia cells to respond to angiogenic signals from endothelial cells suggests a close relationship between the two cell types. In fact, AML cells from patients have been reported to express VEGF and VEGFRs [6, 13, 18]. Fiedler et al. were the first to report that primary AML cells can express VEGF, VEGFR1, and VEGFR2. A greater proportion of AML patients had disease that expressed VEGFR1 compared to VEGFR2 [6]. Padro et al. also screened AML patients, but found that VEGFR2 was more commonly expressed on AML myeloblasts [19]. Together these data support the notion that AML blasts can exhibit a hybrid EC phenotype and that the coexpression of EC surface proteins is variable. The variability of VEGFR expression on AML cells introduces the possibility of a personalized medicine approach to treating AML. For example, in patients who have AML that expresses VEGFRs, treatment with anti-VEGF agents may bring about improved outcomes. 

Given that AML cells can secrete VEGF and express VEGFRs, it stands to reason that AML cells may benefit from an autocrine loop. Indeed, investigators have reported a possible autocrine loop including VEGF produced by leukemia cells and their own VEGF receptors. In particular, VEGFR2+ AML cells were treated with VEGFR2 neutralizing antibodies in serum-free growth conditions. Blocking VEGFR2 resulted in decreased leukemic cell growth, supporting the notion of a VEGF/VEGFR2 autocrine loop [20].

The angiopoietin/Tie2 axis represents another potential autocrine loop in AML. We and others have found that certain myeloid leukemia cell lines, namely, K-562, express angiopoietin-1 (Ang-1), Ang-2, and their receptor, Tie2. Primary human AML specimens have also been reported to express these endothelial cell associated factors and receptor [21]. An *in vitro *study by Reikvam et al. found that blocking angiopoietin interactions with the Tie2 receptor using antibodies led to marked decreases in AML cell proliferation [22]. Interestingly, they found that some primary AML specimens were dependent on autocrine stimulation in order to proliferate, whereas others that did express the angiopoietins and Tie2 proliferated independently of autocrine stimulation. Yet to be defined is the effect of angiopoietin peptibodies such as AMG386 (Genetech) in hematologic malignancies. Clinical studies have begun using these peptibodies in prostate and ovarian cancers [23].

Another explanation for AML myeloblasts that coexpress EC phenotype is the possibility of fusion. Studies by Skinner et al. [24] further explored this idea using an *in vivo* model of primary human AML cells into immunocompromised mice. Hepatic sinusoids were lined by hybrid human AML-murine ECs (AML-EC). Moreover, these fused AML-ECs contained separated human DNA and murine DNA typically found in syncytia. Yet to be determined is whether the expression of EC proteins endows AML myeloblasts with resistance to chemotherapy. If AML-EC hybrids are more resistant to chemotherapy and can regenerate and proliferate AML cell population, then these cells may represent sources of refractory and relapsed disease. 

## 4. Leukemia Hemangioblast Activity: Leukemia Cell Differentiation into Endothelial Cells

Blood and blood vessels are closely linked in developmental biology. In the embryo, the hematopoietic and endothelial lineages are generated from a common mesodermal progenitor, the hemangioblast [25, 26]. Our group and others have demonstrated that adult hematopoietic stem and progenitor cells also exhibit this hemangioblast activity [27–30]. Given that AML cells arise from malignant hematopoietic stem and progenitor cells, it is therefore possible that there may also be a leukemia hemangioblast—generating both malignant leukocytes and malignant ECs.

In one report, a subpopulation of vascular progenitor cells (VEGFR2+ CD31^−^ CD34^−^) harboring the *BCR/ABL* gene fusion was identified in the BM of patients with CML [31]. These cells possessed the potential to form malignant hematopoietic and endothelial cells *in vitro* at the single-cell level. Moreover, when transplanted into NOD/scid mice, these VEGFR2^+^ CD31^−^ CD34^−^ cells were capable of reproducibly transferring CML to transplanted mice and generating ECs within blood vessels that expressed *BCR/ABL*. In other studies, it was shown that transformed genotypes including *BCR/ABL* and the Janus kinase 2 (JAK2) V617F mutation are not readily found in colonies generated in endothelial colony forming cell (ECFC) culture conditions, whereas angiogenic monocytes that form CFU-Hill colonies can harbor such mutations [32, 33]. Together, these results demonstrate the existence of an adult hemangioblast population even in settings of hematological malignancies; however, these data also suggest that ECs harboring cytogenetic mutations may not be derived from putative endothelial progenitor cells (EPCs), which are defined by their ability to form ECFC colonies, but instead from a population of hematopoietic-derived cells. 

Certainly, the existence of a bipotential malignant hematopoietic stem cell could explain a source of relapsed disease and would represent a new target for therapy.

## 5. Potential Novel Therapeutic Approaches to Targeting Leukemia and Endothelial Cell Interactions

The importance of angiogenesis in AML has led to clinical studies of vascular targeting agents in patients with AML (Table 1). Both angio-inhibitory and vascular disrupting strategies are being studied.

Given the presence of VEGF in AML and signs of increased angiogenesis in the bone marrow, investigators have tested anti-VEGF strategies for the treatment of AML. Bevacizumab (anti-VEGF-A antibodies, Avastin) brings about modest clinical efficacy in the treatment of colon cancer when combined with cytotoxic chemotherapy [45]. In the case of AML, bevacizumab was proposed to inhibit leukemia cells by two proposed mechanisms: first, when considering that AML myeloblasts can express VEGF isoforms and VEGFR2, anti-VEGF therapies may have a direct inhibitory effect on malignant myeloblasts. Second, VEGF is an important factor in the angiogenesis of the leukemia niche. Therefore, it was reasoned that bevacizumab may result in AML regression in patients. Two clinical studies of bevacizumab in relapsed and refractory AML have been reported [34, 35]. Dr. Karp and colleagues at Johns Hopkins conducted a phase II clinical study of bevacizumab using a timed sequential therapy approach in 48 patients with either relapsed or refractory AML. Cytarabine was administered on days1–4, followed by 40–60 minutes of mitoxantrone, and finally bevacizumab on day 8. Serum VEGF levels were elevated prior to bevacizumab infusion on day 8 and decreased markedly after infusion. The clinical outcomes of this time sequential therapy showed complete response in 33% of patients, partial response in 15%, and no response in 35%. In the 33% of patients with complete response, the median disease-free survival was about 7 months. These clinical outcomes in a heavily pretreated group are higher than expected with chemotherapy alone and suggest an additive effect of bevacizumab in remitting disease. In the second study, Mesters and colleagues assessed the activity and efficacy of single agent bevacizumab in a small trial of 9 patients with relapsed and refractory AML. In their study, VEGF expression in the bone marrow was decreased after bevacizumab; however, there was no decrease in VEGFR2 and VEGFR2y, suggesting little inhibition of their phosphorylation activity. Furthermore, despite reduced VEGF expression in the bone marrow, there was no significant decrease in blast count after bevacizumab monotherapy. Indeed, we have found similar findings of minimal benefit in preclinical models of AML [15]. Overall, there is low enthusiasm for single agent bevacizumab in AML. However, anti-VEGF-A antibodies may be useful in combination with other chemotherapeutic or vascular disrupting agents.

Another agent that binds and targets the VEGF ligand, and with higher affinity than bevacizumab, is aflibercept VEGF trap. This fusion protein binds to VEGF-A, VEGF-B, and placental growth factor (PlGF). Aflibercept has only been evaluated in solid tumors for tolerability and shows modest antitumor activity [36]. In AML, the study of aflibercept has been restricted to human AML through *in vitro *and mouse xenograft models. The preliminary *in vivo* studies of Lal et al. showed that aflibercept slowed disease progression in two systemic human AML mouse xenograft models. Combining aflibercept with doxorubicin enhanced antileukemia effects, decreased microvessels, and induced perivascular apoptosis [46]. 

Another strategy in targeting VEGF activity is to target VEGFRs. In specific, small molecule tyrosine kinase inhibitors (TKIs) have been designed to impair VEGFR phosphorylation activity. 

Vatalanib, active against VEGFRs and PDGFRs, is well tolerated and has shown clinical activity in multiple solid tumors. Vatalanib has been tested in AML and MDS. A two-armed phase I clinical trial of dose-escalated vatalanib showed that the agent is well tolerated in AML and MDS patients with minimal side effects [43]. Hypertension was reported and mitigated by medical management. A vatalanib dose of 750 mg by mouth daily was established as the maximum tolerated dose (MTD). While the safety profile for this TKI is favorable, the clinical efficacy of Arm 1 monotherapy showed no significant response to treatment, with the greatest efficacy in 2 patients that had prolonged disease stabilization. Combination therapy resulted in 5 complete remission events. This study supports the recurring theme of minimal efficacy in terms of monotherapy small-molecule TKIs in the treatment of AML. 

Cediranib, which was designed with even greater affinity for VEGFR-1 and VEGFR-2, has also shown clinical activity in certain solid tumors such as glioblastoma multiforme and nonsmall cell lung cancer [47, 48]. This TKI also inhibits receptor signaling at nanomolar ranges against c-kit, PDGFR-*β*, and VEGFR-3. In AML, a phase I clinical study of cediranib in 35 leukemia patients showed a correlation between cediranib exposure and plasma VEGF levels and dose- and time-dependent reductions of soluble VEGFR-2 [37]. Also, although there was no connection between clinical activity and microvessel density from treatment, the majority of patients who received the maximum tolerated dose (30 mg/day) did show significant decreases in their bone marrow MVD. In terms of clinical response, only modest benefit was reported. Only four patients out of 31 evaluable subjects showed an objective response. Taking this into consideration, future clinical studies will consider cediranib at 20 mg and 30 mg in combination with standard induction chemotherapies to improve clinical efficacy. 

In AML, activating mutations in FLT3, especially internal tandem duplications, predict for a higher chance for refractory and relapsed disease. Thus, the current standard of care is to refer patients with FLT3 mutant AML for allogeneic hematopoietic cell transplant (allo-HCT), as this is the only potential for cure. However, few older AML patients are candidates for allo-HCT because of comorbidities, difficulties in finding a donor and financial/insurance reasons. Therefore, attempts have been made to target FLT3 activity with inhibitors. 

Sorafenib, a TKI designed to target Ras-Raf/MEK/ERK signaling, but also targets FLT3, has shown clinical activity in renal cell carcinoma and hepatocellular carcinoma [49, 50]. Given that approximately 30% of AML patients have an activating FLT3 mutation, sorafenib was recently tested in AML patients to establish feasibility [39]. Metzelder et al. administered sorafenib in 8 AML patients (FLT3 mutant) between 2007 and 2010. All patients showed rapid hematological responses and complete molecular remissions were observed. The study is ongoing and longer followup is needed and planned [40]. On a much larger scale, Serve et al. conducted a multicenter, randomized, placebo-controlled, double-blind trial of this sorafenib in AML. Investigators administered oral sorafenib versus placebo in combination with standard induction chemotherapy (seven days of cytarabine and three days of idarubicin chemotherapy followed by two cycles of intermediate dose AraC consolidation therapy in 197 AML patients over the age of 60 [41]). One hundred two patients received sorafenib (400 mg daily) and 95 patients received placebo. Hand-foot-skin reactions which were commonly seen in early phase solid tumor trials were also observed in a few AML patients (*n* = 5) receiving sorafenib. Prior to the consolidation AraC cycles, there was a trend in slower regeneration of leukocytes and thrombocytes in the sorafenib arm. However, in terms of clinical response, there were no improvements in event-free survival and overall survival compared to the placebo group. This trial suggests that while targeting FLT3 with sorafenib is tolerable in AML, it shows little clinical activity.

Sunitinib (SU11248) is another angio-inhibitory TKI that has been tested as an antineoplastic agent [37]. In addition to targeting c-Kit, VEGFRs, and PDGFRs, sunitinib also inhibits FLT3. This inhibition profile, therefore, made sunitinib an attractive agent for AML—especially for patients with high risk FLT3 activating mutation. In a phase I study of 15 patients with refractory AML, patients received sunitinib 50 mg daily. No dose limiting toxicities occurred. Adverse events were limited to grade 2 edema, fatigue, and oral ulcerations. There were two fatal hemorrhages, which were potentially related to underlying disease. Escalating the dose to 75 mg resulted in grade 4 toxicities of fatigue, hypertension, and cardiac failure and led to the abandonment of the dose level. In terms of efficacy, there were only partial responses of short duration. Levels of both plasma VEGF and plasma FLT3 ligand (FL) significantly increased from baseline in most of the 16 evaluated patients with no correlation to clinical responses. The significance of VEGF plasma level increases has been reported in other clinical studies and may owe itself to the hypoxic induction of VEGF, whereas the significance of FL has yet to be determined. Given the adverse events at low doses of sunitinib and minimal clinical response, there is low enthusiasm to continue testing this TKI as a monotherapy agent. However, this multitarget agent may enhance response to other agents such as chemotherapy and/or vascular disrupting agents.

The small-molecule TKI semaxanib (SU5416) targets the common VEGF receptors 1 and 2, cKit, and FLT3. In a multicenter phase 2 trial of semaxanib, 42 patients with advanced, c-kit positive AML, either refractory or elderly patients not fit for intensive induction chemotherapy, received at least one dose of treatment [38]. At a dose of 145 mg/m^2^ twice a week, the drug was well tolerated, with mostly mild to moderately severe adverse events. The most striking adverse event, not seen in solid tumor studies of semaxanib, was severe bone pain, which may be attributed to the activity of the drug in bone marrow. Of 25 patients who were evaluable for clinical response, 1 patient achieved a morphological response followed by a relapse after 8 weeks, and 7 of the 25 patients achieved partial response with decreases in bone marrow and peripheral blood leukemic blasts of at least 50%. In terms of biological response, VEGF levels and bone marrow MVD correlated with each other and decreased after semaxanib treatment. Again, the modest clinical activity of this small-molecule TKI dampens enthusiasm for monotherapy in AML. However, the agent did show biological activity and this merits further study, including combination therapy.

Due to their multitargeted potential, TKIs could open the door to more personalized cancer treatments. However, many are metabolized through hepatic cytochrome P450 enzymes and have the potential to interact with many medications commonly prescribed to patients with AML (e.g., antifungal agents such as azoles and chemotherapy). It is conceivable that in the future, AML cell surface expression or genetic mutation may dictate what type of TKI to prescribe. This is particularly attractive in older patients with AML who may be ineligible for high intensity chemotherapy. These older patients may fare better with an oral TKI and low intensity chemotherapy. 

Our own studies have led further efforts to develop multitargeted therapies. Our lab has identified a promising multi-target antivascular treatment strategy, using a novel endothelial cell targeting agent, combretastatin A-1 (OXi4503) [15], alone and in combination with bevacizumab. Combretastatins were discovered in the 1970s from the South African Bush Willow. These agents are structurally similar to colchicine and, like colchicine, bind to *β*-tubulin, lead to microtubule depolymerization and selectively target rapidly proliferating ECs. Unlike colchicine, combretastatins exhibit vascular disruption below maximum tolerated dose (MTD). Therefore, these agents are highly attractive for clinical translation. We and others have shown that naturally occurring combretastatins, CA4 and CA1, potently regress AML in xenograft models. We also showed that the combretastatin CA1 results in a VEGF-driven reactive angiogenesis which supports disease relapse. Therefore we devised a strategy to combine combretastatin (CA1) and anti-VEGF antibodies (bevacizumab). This novel strategy resulted in potent regression of AML. We therefore translated this work into a phase I clinical study of CA1 (OXi4503) in patients with relapsed and refractory AML and MDS (ClinicalTrials.gov Identifier NCT01085656). After establishing MTD, we plan to combine combretastatins with other angio-inhibitor agents like bevacizumab in future clinical trials.

## 6. Summary and Future Directions

Whereas cancer angiogenesis is classically thought of in context to solid tumors, there is mounting evidence that angiogenesis is also significant in leukemia. With specific regard to leukemia and endothelial cells, there are several aspects to consider. First, endothelial cells can support leukemia cells via secreted factors. Whereas a few key axes have been identified (e.g., VEGF/VEGFR and Ang-1/2/Tie2), the full panoply of secreted factors has yet to be defined. Second, leukemia cells can promote endothelial cells. The observation of a codependent relationship creating a vicious cycle of support substantiates the strategy of using endothelial cell targeting agents such as combretastatins. Third, studies identifying critical adhesion molecules between leukemia and endothelial cells are lacking, and this represents an open area of research. These studies will enlighten us of how leukemia cells enter and exit the bone marrow. Understanding how sinusoidal endothelial cells—gatekeeper cells of the marrow—regulate emigration and immigration will lead to novel strategies for mobilizing leukemia out of protective niches and towards heightened sensitivity to treatment. Fourth, leukemia cells can coexpress endothelial cell features. How these features impact sensitivity and protection from conventional treatment need to be better elucidated. Part of these studies will involve inhibiting endothelial cell-associated expression, and it will be important to determine whether this inhibition results in increased sensitivity to treatment. If so, then this would represent a novel strategy for sensitizing leukemia-EC hybrids to treatment. Fifth, the vascular niche in the bone marrow contains many other cell types. Two in particular, perivascular pericytes and intercalated megakaryocytes, participate in the way that the sinusoidal vessels regulate hematopoietic stem and progenitor cell function. Therefore, it is reasonable to consider these other cells types as important players in the governance of leukemia behavior. Finally, beyond cell types, there are many noncellular elements of the bone marrow microenvironment that need to be assessed. These bone marrow conditions include chronic physiological hypoxia, low pH, low glucose levels, and extracellular matrices. All of these factors affect angiogenesis and vice versa; therefore, these factors need to be defined in the context of leukemia pathology to have a more complete picture of how the disease develops, responds to treatment, and relapses.

One important clinical translation of a better understanding of the relationship between angiogenesis and leukemia is in the realm of improved diagnostics. Currently bone marrow biopsies and repeated blood draws are the mainstay for diagnosis, prognosis, and response assessment. With advent of new biologics that have antiangiogenic and antivascular activity, there is a calling for novel biomarkers and methods to measure response and predict for responders. Through the development of a pre-treatment biomarker screening method, we may be able to utilize existing and future antiangiogenic agents to their full potential on a more personalized basis. 

Another clinical translational consideration is how to assess treatment response after administering vascular targeting agents in AML. Currently, there are no established methods. Consider that the primary target is bone marrow blood vessels, it would reason to follow that changes in bone marrow vascularity indicate treatment response. However, there are several methods to quantify bone marrow vascular activity. Serial bone marrow biopsies for measurement of microvessel density, EC function, and angiogenic cytokinesare involve procedures that are painful and possibly affected by sampling area. Furthermore, histologic and biochemical testing takes time and do not provide results in real time. Another method to monitor bone marrow blood vessel activity is via serial computed tomography (CT) scans. However, this method exposes the patient to harmful ionizing irradiation and intravenous contrast carries the risk for nephrotoxic reactions. Magnetic resonance imaging (MRI) is another method to measure changes in bone marrow blood vessels. This method is rapid and does not deliver harmful ionizing irradiation. Moreover, the risks of a contrast (gadolinium) toxicity is lower than with CT scans. Recently, two groups have shown early data that dynamic contrast enhanced- (DCE-) MRI can be used to assess bone marrow vascular perfusion and predict for response to chemotherapy [51, 52].

The last clinical translational consideration is that many of the vascular targeting clinical studies in AML have lacked investigation of mechanisms of action. It is assumed that the administered antivascular agents operated through blood vessel targeting mechanisms, but without an accurate measurement of response this theory has yet to be proven. Thus, it is imperative that rationally designed biomarkers be used to evaluate response to vascular targeting agents. In addition, AML cells themselves can aberrantly express an endothelial-like phenotype, and therefore may be direct targets of vascular targeting agents.

Finally, defining how leukemia exploits the bone marrow vascular niche may lead to promising new therapeutics. We have already translated the vascular disrupting agent, CA1P (OXi4503), into the clinic, and there are many more on the way. As with many antiangiogenic and antivascular agents, cytopenias have been observed in early clinical studies. This is to be expected considering the crosstalk between angiogenesis and hematopoiesis. Therefore, the application of these novel agents into the leukemia unit and clinic will require careful administration and an “induction” mindset. Blood product transfusions and prophylactic antibiotics will likely be required in these AML patients. However, with adequate support, these agents may show promising results over time.

## Figures and Tables

**Table 1:** Vascular targeting strategies for patients with acute myeloid leukemia.

Agent	References	Target	Phase of clinical study	# of Patients in AML trial(s)	Clinical activity
Bevacizumab	[34, 35]	VEGF-A	Phase 2	48, 9	None as monotherapy; minimal when combined with chemotherapy
Aflibercept	[36]	VEGF-A, VEGF-B, PlGF	Preclinical		
Sunitinib	[37]	VEGFR-1,-2,-3, PDGFRs, c-KIT, FLT3, CSF-1, RET	Phase 1	15	Minimal as monotherapy
Semaxanib	[38]	VEGFR-1, -2, c-KIT, FLT3	Phase 2	6	Minimal as monotherapy
Sorafenib	[39–41]	FLT3, VEGFR-2, -3, PDGFR, Raf, c-KIT	Phase 3	127	None
Axitinib	[42]	VEGFR-1, -2, -3, PDGFR-*β*, c-KIT	Phase 2	12	Minimal as monotherapy
Cediranib	[37]	VEGFR-1, -2, -3, PDGFR-*β*, c-KIT	Phase 1	35	Modest as monotherapy
Vatalinib	[43]	VEGFR-1, -2, -3, PDGFR-*β*, c-KIT, FMS	Phase 1	17	None with monotherapy; minimal when combined with chemotherapy
Combretastatin A-4-Phosphate (Zybrestat)	[44]	Microtubule depolymerization in endothelial cells, direct cytotoxicity to AML cells	Preclinical		
Combretastatin A-1-Phosphate (OXi4503)	[15]	Microtubule depolymerization in endothelial cells, direct cytotoxicity to AML cells	Phase 1	Ongoing	
